# Bromodomains in Human-Immunodeficiency Virus-Associated Neurocognitive Disorders: A Model of Ferroptosis-Induced Neurodegeneration

**DOI:** 10.3389/fnins.2022.904816

**Published:** 2022-05-12

**Authors:** Adonis Sfera, Karina G. Thomas, Christina V. Andronescu, Nyla Jafri, Dan O. Sfera, Sarvin Sasannia, Carlos M. Zapata-Martín del Campo, Jose C. Maldonado

**Affiliations:** ^1^Patton State Hospital, San Bernardino, CA, United States; ^2^Department of Psychiatry, University of California, Riverside, Riverside, CA, United States; ^3^Department of Anthropology, Stanford University, Stanford, CA, United States; ^4^Department of Medicine, Shiraz University, Shiraz, Iran; ^5^Instituto Nacional de Cardiologia Ignacio Chavez, Mexico City, Mexico; ^6^Department of Medicine, The University of Texas Rio Grande Valley, Edinburg, TX, United States

**Keywords:** ferroptosis, neurodegenerative disorders, iron, BRD4, miR-29

## Abstract

Human immunodeficiency virus (HIV)-associated neurocognitive disorders (HAND) comprise a group of illnesses marked by memory and behavioral dysfunction that can occur in up to 50% of HIV patients despite adequate treatment with combination antiretroviral drugs. Iron dyshomeostasis exacerbates HIV-1 infection and plays a major role in Alzheimer’s disease pathogenesis. In addition, persons living with HIV demonstrate a high prevalence of neurodegenerative disorders, indicating that HAND provides a unique opportunity to study ferroptosis in these conditions. Both HIV and combination antiretroviral drugs increase the risk of ferroptosis by augmenting ferritin autophagy at the lysosomal level. As many viruses and their proteins exit host cells through lysosomal exocytosis, ferroptosis-driving molecules, iron, cathepsin B and calcium may be released from these organelles. Neurons and glial cells are highly susceptible to ferroptosis and neurodegeneration that engenders white and gray matter damage. Moreover, iron-activated microglia can engage in the aberrant elimination of viable neurons and synapses, further contributing to ferroptosis-induced neurodegeneration. In this mini review, we take a closer look at the role of iron in the pathogenesis of HAND and neurodegenerative disorders. In addition, we describe an epigenetic compensatory system, comprised of bromodomain-containing protein 4 (BRD4) and microRNA-29, that may counteract ferroptosis by activating cystine/glutamate antiporter, while lowering ferritin autophagy and iron regulatory protein-2. We also discuss potential interventions for lysosomal fitness, including ferroptosis blockers, lysosomal acidification, and cathepsin B inhibitors to achieve desirable therapeutic effects of ferroptosis-induced neurodegeneration.

## Graphical Abstract

Both HIV-1 and cART alter the lysosomes, increasing intracellular iron and the risk of ferroptosis. Dysfunctional lysosomes release the ferroptosis drivers iron, Ca^2+^ and cathepsin B (catB), promoting neuronal and oligodendrocyte loss, reflected in the white and gray matter pathology. The host responds to lysosomal damage by activating an epigenetic axis comprised of bromodomain 4 (BRD4) and microRNA-29 family (miR-29) that promptly suppresses lysosomal function, lowering ferritinophagy. As there is an inverse relationship between miR-29 and BRD4, HIV-1 inhibition of miR-29, upregulates BRD4, blocking ferritinophagy. The BRD4/miR-29 system also inhibits iron regulatory protein-2 (IRP-2) and augments cystine/glutamate antiporter xCT (SLC7A11), lowering the odds of ferroptosis. 
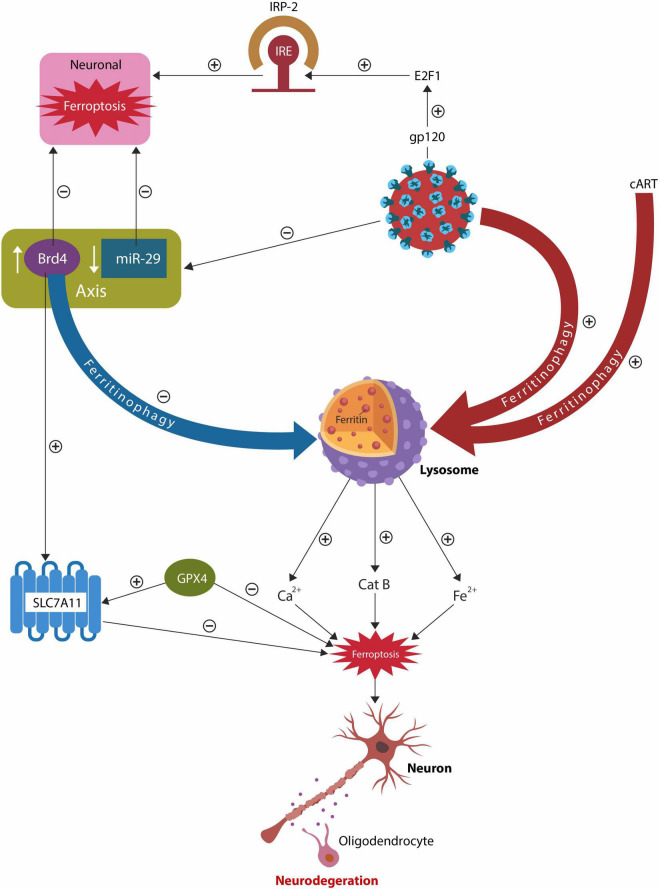

## HIGHLIGHTS

- HIV Patients With HAND Often Develop Early Neurodegeneration and Iron Dysmetyabolism, Likely Implicating Ferroptosis in this pathology.

- Many viruses, including HIV-1, exploit the host endosomal-lysosomal system to acquire iron and egress host cells.

- Both HIV-1 and cART can disrupt the lysosomes, promoting ferroptosis by releasing cathepsin B, iron, and Ca^2+^.

- Dysfunctional lysosomes impair both ferritinophagy and myelin synthesis, increasing the risk of neuronal and glial ferroptosis.

- An epigenetic BRD4/miR-29 system may oppose ferroptosis by boosting SLC7A11 and lowering ferritin autophagy.

## Introduction

HIV-associated neurocognitive disorder (HAND), encountered in up to 50% of HIV patients, is characterized by cognitive deficits that may occur despite adequate treatment with combination antiretroviral therapy (cART) (Ru and Tang, 2017). Although the severity of HAND is lowered by cART, people living with HIV (PLWH), continue to display high rates of cognitive impairment and often develop Alzheimer’s disease (AD) earlier in life compared to the general population (Calcagno et al., 2021; Sharma, 2021). As high intracellular iron worsens HIV-1 prognosis and iron proteins are upregulated in HAND, ferroptosis-induced neurodegeneration (FIN) may contribute to this disorder (Patton et al., 2017; Milanini et al., 2019).

Viruses require iron for replication and often obtain this nutrient by targeting the iron-rich organelles, mitochondria, and lysosomes, disrupting their function, including ferritinophagy and myelination (Chang et al., 2015).

Ferroptosis is a programmed cell death triggered by iron-mediated lipid peroxidation in the absence of antioxidants glutathione (GSH) or glutathione peroxidase 4 (GPX4). Under normal circumstances, iron is stored in ferritin, a protein that undergoes lysosomal autophagy to release this biometal as needed. Dysfunctional ferritinophagy triggers toxic oxidative stress by upregulating intracellular iron, promoting pathology, including neurodegeneration. Aside from iron, ferroptosis can be triggered by low uptake of cysteine or glutamine via SLC7A11, an amino acid transporter specific for cysteine and glutamate, as well as the loss of GPX4 (Tang et al., 2021). Viral infections associated with increased iron absorption or upregulation of intracellular iron are likely to result in ferroptosis. Ferroptotic cell death is characterized by the release of damage-associated molecular patterns (DAMPs) that trigger immunogenicity and neuroinflammation, hallmarks of both HAND and AD (Smail and Brew, 2018; Sun et al., 2018). Lysosomes, the master regulators of iron metabolism, control ferroptosis via ferritin autophagy (ferritinophagy), a process characterized by iron release (Rizzollo et al., 2021). Many viruses hijack the endosomal-lysosomal system (ELS) to acquire iron, precipitating ferroptosis and FIN (Gao et al., 2017).

Recent studies have found that both cART and the HIV-1 antigen, trans-activator of transcription (Tat), alter the host ELS, upregulating ferritinophagy and iron release (Fredericksen et al., 2002; Hui et al., 2012; Tripathi et al., 2019; Cao et al., 2021) (Graphical Abstract). In addition, virus or cART-induced lysosomal dysfunction can activate microglial cells that often eliminate healthy neurons and synapses, further contributing to neurodegeneration (Tripathi et al., 2019; Kapralov et al., 2020; Cao et al., 2021; Miyanishi et al., 2021). Microglia are highly susceptible to ferroptosis and harbor latent HIV-1, therefore ferroptotic disintegration of these cells release DAMPs, triggering neuroinflammation (Lisi et al., 2016; Chivero et al., 2017; Shankaran et al., 2017; Wallet et al., 2019; Borrajo et al., 2021). Indeed, iron-activated microglia and macrophages, documented in HIV-1 infection, are believed to drive HAND pathology (Boelaert et al., 1996; Kenkhuis et al., 2021). In addition, microglia release cathepsin B (CatB), a protein associated with premature brain aging, neurotoxicity, and the accumulation of pathological, hyperphosphorylated Tau (pTau) (von Bernhardi et al., 2015; Nakanishi, 2020). Interestingly, as CatB possesses endopeptidase activity, the SARS-CoV-2 virus hijacks this protein to activate the S (spike) antigen, increasing infectivity (Schornberg et al., 2006; Mitrović et al., 2016; Huang et al., 2020; Padmanabhan et al., 2020). Moreover, individuals with HIV-1 infection on long-term cART present with higher brain deposition of pTau, linking this virus and its treatment to the risk of developing tauopathies (Anthony et al., 2006; Brown et al., 2014; Rao and Adlard, 2018; Mangan, 2021). These findings indicate that HIV-1 and CART induce lysosomal damage and predispose to FIN as elevated intracellular iron increases the odds of lipid peroxidation and ferroptotic cell death (Jiang et al., 2021).

**FIGURE 1:**
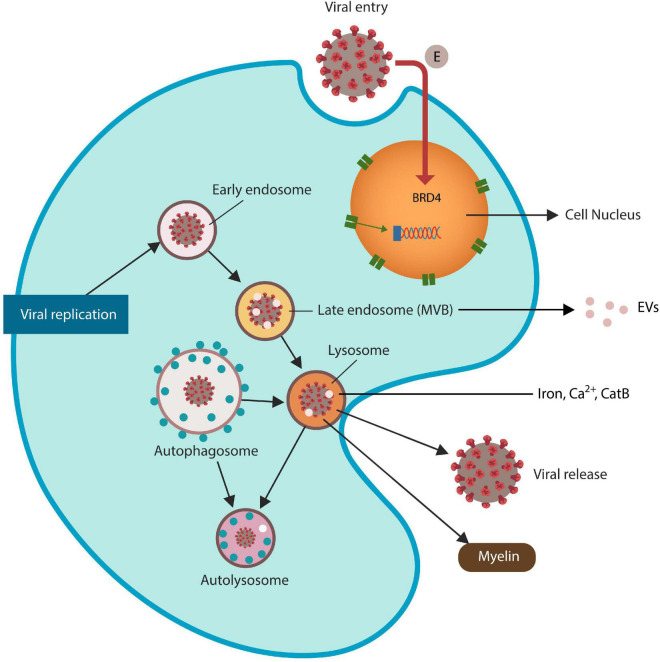
SARS-CoV-2 virus and HIV Tat antigen ingress host cells via ELS. The SARS-CoV-2 envelope (E) protein is a direct inhibitor of BRD4, increasing the risk of ferroptosis. Viruses that exploit ELS to egress host cells may disrupt lysosomal exocytosis of myelin and Tau protein (not shown). Late endosomes generate extracellular vesicles (EVs) that can spread viral proteins to the neighboring cells. Dysfunctional lysosomes may “leak” ferroptosis-driving molecules, including iron, Ca^2+^ and CatB, contributing to ferroptosis-induced neurodegeneration (FIN).

Lysosomes are iron-rich subcellular organelles specialized in the degradation of proteins derived from autophagy, endocytosis, and phagocytosis (Platt et al., 2012; Figure 1). Aside from recycling endogenous biomolecule, autophagy also eliminates malignant and virus-infected cells, actively participating in host immunity (Choi et al., 2018). Many viruses, including SARS-CoV-2 and HIV Tat protein exploit the ELS to enter and exit host cells, disrupting this pathway and predisposing to FIN (Fan and He, 2016; Khan et al., 2020; Chen D. et al., 2021). Indeed, viruses that exploit the lysosome alter local pH and membrane permeability, facilitating the release of ferroptosis drivers iron, calcium (Ca^2+^) and CatB (Hui et al., 2012; Gorshkov et al., 2021; Nagakannan et al., 2021; Pedrera et al., 2021). For example, recent studies linked cytosolic Ca^2+^ upregulation to both ferroptosis and excitotoxicity, connecting the two metals to cell death (Gleitze et al., 2021; Pedrera et al., 2021). In addition, HIV has been reported to generate massive reactive oxygen and nitrogen species (RONS) associated with HAND, likely by aberrant microglial activation (Borrajo et al., 2021).

Recent studies have reported that lysosomal exocytosis is required for oligodendrocytes (OLGc) and Schwan cells myelination, suggesting that dysfunctional ELS could lead to white matter damage in HAND and AD (Shen et al., 2016). Others have connected dysfunctional ELS with the dissemination and seeding of pTau, further implicating this system in tauopathies (Tanaka et al., 2019; Jiang and Bhaskar, 2020; Polanco et al., 2021; Sebastián-Serrano et al., 2022).

In this mini review, we take a closer look at the virus induced ELS dysfunction in the pathogenesis of HAND and neurodegenerative disorders. In addition, we propose that FIN, triggered by HIV-1 infection and long-term cART use, may be counteracted by an epigenetic system comprised of bromodomain protein 4 (BRD4) and microRNA-29 (miR 29) that inhibits ferritinophagy by several mechanisms, including direct antiviral action, lysosomal suppression, SLC7A11 activation, and iron regulatory protein-2 (IRP-2) inhibition. We also discuss potential interventions for lysosomal fitness, including V-ATPase inhibitors, ferroptosis blockers, and CatB inhibitors.

## Ferroptosis and the Endosomal-Lysosomal System

The ELS is comprised of intracellular vesicular compartments, including early endosomes, recycling endosomes, and late endosomes [also called multivesicular bodies (MVBs)] that merge with lysosomes. Autophagosomes also join the lysosomes to recycle their cargoes (Figure 1). Many viruses, including HIV-1 and SARS-CoV-2, acquire iron by usurping the lysosome, a process that upregulates ferritinophagy, iron release and the risk of FIN (Hui et al., 2012; Chauhan and Khandkar, 2015; Hu et al., 2015; Yambire et al., 2019; Blaess et al., 2020; Figure 1).

As autophagy controls viral infections by catabolizing infected cells along with the virus, many viruses have developed the ability to evade immunity by manipulating autophagy. For example, HIV-1 hijacks the ELS by Tat, Nef, and ENV antigens interaction with the autophagy proteins or mammalian target of rapamycin (mTor) components (Nardacci et al., 2017).

Lysosomal exocytosis, a mechanism of content secretion into the extracellular space, is mediated by lysosomal fusion with the cell plasma membrane that enables cargo release. HIV-1 and SARS-CoV-2 hijack the ELS to enter and egress host cells, disrupting vesicular homeostasis, including lysosomal exocytosis, and local pH (Hui et al., 2012; Nardacci et al., 2017; Blaess et al., 2020). Recent studies have reported that myelin biosynthesis requires adequate lysosomal exocytosis, suggesting that dysfunctional ELS may lead to both gray and white matter pathology (Chen D. et al., 2021; Kreher et al., 2021). Indeed, viruses that exit host cells through the lysosomes may disrupt myelination, promoting HAND and neurodegenerative disorders (Jensen et al., 2015; Buratta et al., 2020). Along these lines, novel neuroimaging studies have detected white matter changes in the prodromal phase of AD [prior to the development of pTau or beta-amyloid (Aβ)], indicating that myelin pathology is more common in his disorder than previously thought (Nasrabady et al., 2018; Pichet Binette et al., 2021). Moreover, a growing body of evidence has demonstrated myelin breakdown and dysfunctional OLGc progenitor cells (OPCs) in HAND and AD, linking both conditions to white matter pathology (Kimura-Kuroda et al., 1994; Bernardo et al., 1997; Lackner et al., 2010; Jensen et al., 2019). Furthermore, as OLGc are the predominant iron-containing cells in the brain and highly susceptible to ferroptosis, their demise may increase local iron, predisposing to FIN (Nobuta et al., 2019; Jhelum et al., 2020). Also, the ELS-released pTau, iron, Ca^2+^, CatB, and myelin maintain the neurodegenerative and HAND pathology (Joy et al., 2010; Chen et al., 2012; Xu Y. et al., 2021).

Recent studies found that elevated intracellular iron increases pTau and its aggregation, linking tauopathies to dysfunctional ELS (Brown et al., 2014; Rao and Adlard, 2018). In addition, iron dyshomeostasis and excessive pTau, documented in HAND, AD, and traumatic brain injury (TBI), implicate ELS in these pathologies (Canzoniero and Snider, 2005; Ravi et al., 2016; Cantres-Rosario et al., 2019; Hook et al., 2020; Nagakannan et al., 2021; Pedrera et al., 2021). Furthermore, as pTau acts as a cell-penetrating peptide, it may trigger cell-cell fusion and senescence, probably accounting for the accelerated aging and early development of AD in PLWH on long term cART (Ferrell and Giunta, 2014; Veloria et al., 2017; Osorio et al., 2022). Along these lines, HIV infected macrophages were shown to enter the brain and secrete neurotoxic CatB, triggering premature senescence, ferroptosis and FIN (Cantres-Rosario et al., 2019). Excessive CatB was also associated with cancer, connecting ELS dysfunction to tumorigenesis and metastases (Gondi and Rao, 2013; Ruan et al., 2015). On the other hand, CatB inhibitors may avert HAND, TBI, and cancer (Ha et al., 2012; Kos et al., 2014; Hook et al., 2015; Zenón-Meléndez et al., 2022). Cathepsin B was also associated with autoimmune disorders and osteoporosis, suggesting that a better understanding of this protein and its inhibitors could improve the treatment of several disorders that lack specific therapies (Toomey et al., 2014; Li et al., 2017; Ansari et al., 2022). In addition, several antipsychotic agents possess anticancer and antiviral properties, suggesting that these pathologies may intersect at the ELS level (Daniel et al., 2001; Kuzu et al., 2017; Girgis and Lieberman, 2021; Lu et al., 2021) (discussed in the section Psychotropic drugs). Other novel studies show that viral infections may precipitate ferroptosis by hijacking Ca^2+^ channels and pumps, suggesting a role for calcium channel blockers in the treatment of viral infections (Chen et al., 2019; Jayaseelan and Paramasivam, 2020; Straus et al., 2021). Indeed, as HIV Tat antigen upregulates pTau, it likely promotes FIN (Tripathi et al., 2016; Majerníková et al., 2021; Wang et al., 2022). Moreover, HIV infection was shown to increase apolipoprotein ε4 (ApoE4), an iron-upregulated biomolecule and AD risk factor, suggesting FIN involvement (Ayton et al., 2015; Diouf et al., 2019; Kagerer et al., 2020). Furthermore, as viruses upregulate both ApoE4 and iron, they may be capable of triggering FIN directly (Corder et al., 1998; Burt et al., 2008; Kuo et al., 2020; Gkouskou et al., 2021; Yim et al., 2022).

Taken together, ELS is situated at the crossroad of viral infections, cancer, and neuropsychiatric illness, probably explaining the beneficial effect of lysosomal therapeutics in these pathologies.

## The BRD4/MiR-29 Compensatory System

Bromodomains are chromatin-associated molecules that interact with acetylated lysine residues on histone proteins, regulating numerous cellular processes, including replication, genome repair and the autophagic lysosomal function (Mujtaba et al., 2007; Fujisawa and Filippakopoulos, 2017; Sakamaki et al., 2017; Li et al., 2018). BRD4 is an epigenetic reader that regulates gene expression by forming a complex with the positive transcription elongation factor b (P-TEFb), promoting RNA polymerase II (Pol II), a mediator of DNA-dependent RNA synthesis (Jung et al., 2014). Novel studies have implicated BRD4 in various pathologies, ranging from inflammation, to cancer, CNS, and viral diseases (Zhu et al., 2012; Korb et al., 2015; Hajmirza et al., 2018). In preclinical studies, bromodomain and extra-terminal motif (BET) inhibitors (BETis) were shown to limit the progression of several cancers, rendering this protein a pharmacological target.

Recently, however, pan-BRD4 blockers, such as JQ1, were found detrimental as they trigger ferroptosis, damaging the genome, immunity and white matter (Chiu et al., 2017; Donati et al., 2018; Mita and Mita, 2020). On the other hand, domain-specific BETis appear to have fewer adverse effects and some have already been approved for clinical use (Pérez-Salvia and Esteller, 2017; Petretich et al., 2020). BRD4 negatively regulates ferroptosis by activating xCT, repairing the DNA, and inducing senescence-associated secretory phenotype (SASP), a ferroptosis resistant cellular program (Tasdemir et al., 2016; Sui et al., 2019; Lam et al., 2020; Tang et al., 2022). In addition, BRD4 promotes iron sequestration in ferritin to withhold it from pathogens, likely implicating this protein in nutritional immunity (Sui et al., 2019). Moreover, BRD4 displays direct antiviral properties, including inhibition of HIV Tat antigen, hence viruses must neutralize this protein to thrive (Wang et al., 2020; Alamer et al., 2021; Xu X. et al., 2021; Table 1). Indeed, several viruses, including HIV and SARS-CoV-2 have developed the ability to usurp BRD4, overcoming nutritional immunity (Chen I. P. et al., 2021). For example, the E (envelope) protein of SARS-CoV-2 virus inhibits BRD4, neutralizing the function of this epigenetic reader (Gordon et al., 2020). As BRD4 represses lysosomal autophagy, including ferritinophagy, viral hijacking of this protein may directly induce ferroptosis (Sakamaki et al., 2017). Moreover, BRD4 protects mitochondria by safeguarding the transcription of mitochondrial genes located in the nucleus (Kim et al., 2020). This is significant as earlier studies have implicated mitochondria and BRD4 in memory formation, suggesting a direct mechanism for virus-mediated neurodegeneration (Korb et al., 2015; Khacho et al., 2017; Zhang et al., 2022).

**TABLE 1:** Anti-FIN properties of BRD4/MiR-29.

BRD4/miR-29 neuroprotective properties	References
Antiviral effect	Frattari et al., 2017; Wang et al., 2020; Alamer et al., 2021; Xu X. et al., 2021
HIV Tat protein inhibition	Zhu et al., 2012; Huang et al., 2017
Decrease ferritinophagy	Shimizu et al., 2007; Sui et al., 2019
Improve mitochondrial function	Kim et al., 2020
Decrease HIV latency	Frattari et al., 2017
Upregulate xCT (SLC7A11)	Tasdemir et al., 2016; Tang et al., 2022
Genome repair	Lam et al., 2020
Promotes SASP	Tasdemir et al., 2016
Promotes iron sequestration	Sui et al., 2019

MicroRNAs (miRs) are non-coding ribonucleic acids (RNAs) that regulate gene expression by interacting with the 3′ untranslated region (3′ UTR) of target mRNAs (O’Brien et al., 2018). MiR-29 family, comprised of miR-29a, miR-29b, and miR-29c, displays antiviral properties, including inhibition of HIV-1 Nef protein (Ahluwalia et al., 2008; Adoro et al., 2015; Monteleone et al., 2015). In addition, IL-21/miR-29 axis was associated with HIV-1 latency, suggesting that enhancing this pathway may eradicate the virus from reservoirs (Frattari et al., 2017). Indeed, to counteract its antiviral action, HIV-1 has developed the ability to inhibit miR-29 via Tat antigen, promoting viral latency (Bennasser et al., 2005; Ruelas and Greene, 2013; Monteleone et al., 2015). As there is an inverse relationship between miR-29 and BRD4, HIV-1-mediated miR-29 downregulation lowers ferritinophagy by increasing BRD4 (Kohnken et al., 2018; Huang et al., 2019). Recently, miR-29 was found to lower intracellular iron by inhibiting iron regulatory protein 2 (IRP-2), lowering the risk of ferroptosis and FIN (Ripa et al., 2017) (Graphical Abstract). A recent preclinical study associated miR-29 family with gene expression in aging brain, showing that this miR lowers the expression of IRP-2 and the signaling with the iron responsive element (IRE), decreasing the intracellular iron load (Ripa et al., 2017). Opposing this mir-29 action, the HIV-1 glycoprotein 120 (gp 120) activates IRP-2 via E2F transcription factor 1 (E2F1), precipitating ferrocytosis (Shimizu et al., 2007). This is significant as several studies demonstrated low miR-29 expression in AD, linking this miR to the pathogenesis of neurodegenerative disorders and HAND (Lu et al., 2016; Müller et al., 2016; Pereira et al., 2016; Jahangard et al., 2020). Moreover, BRD4 has inhibitory effects on HIV-1 Tat protein, blocking viral latency and indicating that BRD4/miR-29 manipulation could eradicate latent HIV-1 (Zhu et al., 2012; Huang et al., 2017; Table 1).

Taken together, BRD4/miR-29 may comprise an epigenetic system that opposes FIN by several mechanisms, including direct antiviral action, iron sequestration in ferritin, IRP-2 downregulation, and suppression of lysosomal function, including ferritinophagy. As BRD4/miR-29 axis withholds iron from pathogens, we speculate that this system may drive nutritional immunity.

## Potential Interventions

The role of ferroptosis in viral infections is closely connected to the concept of nutritional immunity, intracellular iron sequestration to withhold it from pathogens (Núñez et al., 2018). Although protective against infections, iron sequestration may escalate the risk of ferroptosis as it places this biometal in the proximity of lipids, increasing the risk of peroxidation (Chao et al., 2020; Chen et al., 2020). For this reason, lowering neuronal ferroptosis by upregulating BRD4 may decrease ferritinophagy and neuronal ferroptosis.

In this section, we focus on pharmacological agents that may lower the FIN risk at the ELS level.

## V-Atpase Inhibitors

Under normal circumstances, lysosomal pH must be highly acidic (4.5–5.5) for protein degradation to occur. This is accomplished via vacuolar (H^+^) ATPase (or V-ATPase) that pumps protons into the organelle to lower its pH (Song et al., 2020). Several viruses and their antigens, including HIV-1 Nef protein, usurp V-ATPase, increasing ferritin autophagy and intracellular iron, as well as the risk of ferroptosis and FIN (Lu et al., 1998; Castro-Gonzalez et al., 2021).

Over the past two decades, several V-ATPase inhibitors have been developed, including concanamycin A, bafilomycin A1, saliphenylhalamide, and quinazolines (Garcia-Rodriguez et al., 2015). Quinazolines were the newest addition to the armamentarium of V-ATPase inhibitors. They are small electrophilic molecules that comprise the common denominator of over 150 naturally occurring alkaloids with numerous biological properties (Chen et al., 2017). These agents possess anti-HIV, anticancer and anti-neurodegenerative properties, implicating ELS dysfunction in these pathologies (Colacurcio and Nixon, 2016; Hu et al., 2018; Whitton et al., 2018; Le-Nhat-Thuy et al., 2020). Indeed, the quinazolinone compound, PBT434 possess iron chelating properties suggesting that lowering this biometal may benefit the patients with these conditions (Bailey et al., 2021).

## N-Acetylcysteine

Recent studies have shown that N-acetylcysteine (NAC) can reverse cART-induced microglial activation and inhibit ferritinophagy (Tripathi et al., 2020). NAC is a widely used drug, primarily as an antidote for acetaminophen overdose, but possesses many other beneficial effects, including correcting the oxidative stress-mediated ELS dysfunction, suggesting that it may reverse some neurological complications of HAND (Tripathi et al., 2020). Interestingly, NAC has been evaluated for its senolytic properties against brain aging, indicating potential efficacy against HAND and AD (Tardiolo et al., 2018). In addition, NAC possesses anticancer and anti-HIV, properties, further linking ELS to these pathologies (Roederer et al., 1992; Deng et al., 2019). Moreover, as NAC supplementation increases GSH and GPX, it may also suppress ferroptosis and FIN (Karuppagounder et al., 2018; Hu et al., 2021).

## Aryl-Thiazole Compounds

Aryl-thiazole compounds are novel agents that include N2-[2-chloro-4(3,4,5-trimethoxy phenyl azetidin-1-yl]-N4-(substituted aryl)-1,3-thiazole-2,4-diamine (4a–g), a lipid peroxidation blocker, indicating potential beneficial effect against FIN (Djukic et al., 2018). Aryl-thiazole compounds possess antiviral, anticancer and anti-neurodegenerative properties by modulating γ-secretase (Lu et al., 2009; Dawood et al., 2015; Bhattarai et al., 2021). In addition, (3Z)-3-(2-[4-(aryl)-1,3-thiazol-2-yl]hydrazin-1-ylidene)-2,3-dihydro-1H-indol-2-one presents with reverse transcriptase inhibiting properties, potentially lowering HIV-1 replication (Meleddu et al., 2015).

## Pyridine, Acetamide, and Benzohydrazine

Pyridine, acetamide, and benzohydrazine are CatB inhibitors with antiviral, anti-neurodegenerative and anticancer properties (Prachayasittikul et al., 2017; Alizadeh and Ebrahimzadeh, 2021; Chitranshi et al., 2021). As CatB is a ferroptosis driver, the inhibitors of this cysteine protease may benefit the patients with AD and HAND. In addition, these molecules were shown to modulate the HIV-1 gene expression and inhibit Tat protein, suggesting antiviral properties (Balachandran et al., 2017). Other recently developed CatB inhibitors are nitroxoline (8-hydroxy 5-nitroquinoline) derivatives, inhibitors of endopeptidase and exopeptidase activities of this enzyme. These drugs have established antibiotic, anticancer and anti-neurodegenerative properties (Knez et al., 2015).

## Ferrostatin-1 and Liproxstatin-1

Ferrostatin-1 (Fer-1) and liproxstatin-1 (Lip-1) comprise a new generation of ferroptosis inhibitors that function as radical traps and scavengers of lipid hydroperoxyl groups (Miotto et al., 2020). These agents are potential COVID-19 treatments, while their inhibitors have been studied as ferroptosis inducers in cancer cells (Subburayan et al., 2020; Yang and Lai, 2020). Moreover, Fer-1 and Lip-1 have been evaluated for efficacy against viral infections and neurodegeneration (Chen K. et al., 2021; Wang et al., 2021).

## Iron Chelators

Iron chelation was demonstrated to protect lysosomal membranes against peroxidative injury, lowering the risk of ferroptosis (Kurz et al., 2006). Indeed, iron chelators are currently being assessed for efficacy against neurodegenerative disorders, including AD (Dusek et al., 2016) (NCT03234686). These agents have shown antiviral effects, especially against HIV-1 and SARS-CoV-2, suggesting that withholding iron from pathogens can be an efficient anti-infectious strategy (van Asbeck et al., 2001; Chhabra et al., 2020). By protecting lysosomal membranes, chelation therapy lowers CatB cytosolic “escape” and subsequent neurotoxicity (Lin et al., 2010). In addition, iron chelators have shown beneficial effects in cancer, probably by decreasing mitochondrial energy production and starving the tumors (Fryknäs et al., 2016).

## Chloroquine and Hydroxychloroquine

The anti-malaria drugs, chloroquine (CQ) and hydroxychloroquine (HCQ) are autophagy blockers that accumulate in the ELS, neutralizing luminal pH. These compounds possess antiviral and anticancer properties, probably by blocking lysosomal exocytosis (Neely et al., 2003; Halcrow et al., 2019). Interestingly, recent studies in patients with rheumatoid arthritis (RA) showed that prolonged treatment with CQ and HCQ decreased the risk of Parkinson’s disease (PD), indicating anti-neurodegenerative properties (Paakinaho et al., 2022). Indeed, as CQ and HCQ inhibit CatB, a protein implicated in the pathogenesis of both RA and PD, it is likely that these drugs inhibit CatB (Hashimoto et al., 2001; Tsujimura et al., 2015; Mahoney-Sánchez et al., 2021; Zhao et al., 2022).

## Psychotropic Drugs

Second generation antipsychotic drugs, and several antidepressants were shown to possess antioxidant properties by upregulating GSH, lowering the risk of ferroptosis (Quincozes-Santos et al., 2010; Caruso et al., 2020). In addition, as GSH possesses antiviral properties, psychotropic drugs may enhance autophagy, facilitating the clearance of cancerous and pathogen-infected cells (Fraternale et al., 2006). Indeed, as some psychotropics enter cells via ELS, they may eliminate the viruses they encounter throughout this pathway (Benton et al., 2010; Canfrán-Duque et al., 2016; Schneider et al., 2016; Vela, 2020; Fred et al., 2022). Moreover, as several psychotropic drugs display anticancer properties, they likely inhibit CatB, lowering the cytotoxicity of this protein (Varalda et al., 2020). Furthermore, newly synthesized ferroptosis inhibitors, the phenothiazine analogs, alter autophagy, probably explaining their efficacy against neurodegenerative disorders (Liu et al., 2020; Posso et al., 2022). Interestingly, the fast-acting antidepressant drug, ketamine, was found to upregulate miR-29, linking depression to ferroptosis, suggesting that the BRD4/miR-29 system may have antidepressant effects (Stolke et al., 1980; Paul et al., 2014; Wan et al., 2018).

## Cathepsin B Inhibitors

CatB dysfunction was associated with several pathologies, including autoimmune disorders, cancer, drug addiction, neurodegeneration, and viral infections (Toomey et al., 2014; Li et al., 2017; Ansari et al., 2022). For example, the anthelminthic drug, niclosamide, demonstrates anticancer, antiviral, and neuroprotective properties, likely by inhibiting CatB and restoring ELS homeostasis (Circu et al., 2016; Goulding et al., 2021; Weiss et al., 2021). Indeed, niclosamide was found to inhibit HIV-1 proliferation and activate PTEN-induced kinase 1 (PINK1), indicating potential benefit in neurodegenerative disorders and likely HAND (Niyomdecha et al., 2020; Jang et al., 2022). Interestingly, sigma-1 (Sig1R) agonists, such as BD1047, were shown to downregulate CatB, ameliorating HAND, especially in cocaine users (López et al., 2019). Along this line, fluvoxamine, a potent Sig1R agonist, with antiviral properties may be a yet unknown CatB inhibitor.

## Conclusion

HIV-1 infection and long-term cART, enhance lysosomal ferritinophagy, releasing excessive amounts of iron, Ca^2+^ and CatB that may trigger FIN. The host responds to this insult by activating an epigenetic system comprised of BRD4/miR-29 that blocks dysfunctional lysosomes and iron release via IRP-2 and SLC7A11. As BRD4 is upregulated by low miR-29 and HIV-1 inhibits miR-29, ferritinophagy is downregulated, lowering FIN. In addition, as BRD4 inhibits HIV-1 Tat protein, modulation of BRD4/miR-29 system may eradicate latent HIV-1 from reservoirs, such as microglia and macrophages.

## Author Contributions

All authors listed have made a substantial, direct, and intellectual contribution to the work, and approved it for publication.

## Conflict of Interest

The authors declare that the research was conducted in the absence of any commercial or financial relationships that could be construed as a potential conflict of interest.

## Publisher’s Note

All claims expressed in this article are solely those of the authors and do not necessarily represent those of their affiliated organizations, or those of the publisher, the editors and the reviewers. Any product that may be evaluated in this article, or claim that may be made by its manufacturer, is not guaranteed or endorsed by the publisher.
